# Lipid functionalized silver-coated carbon dot-capped manganese ferrite as drug-free core-shell nanoparticles for multimodal imaging and therapy

**DOI:** 10.5599/admet.2905

**Published:** 2025-08-26

**Authors:** Anbazhagan Thirumalai, Pazhani Durgadevi, Venkatakrishnan Kiran, Koyeli Girigoswami, Alex Daniel Prabhu, Agnishwar Girigoswami

**Affiliations:** 1 Medical Bionanotechnology, Faculty of Allied Health Sciences (FAHS), Chettinad Hospital & Research Institute (CHRI), Chettinad Academy of Research and Education (CARE), Kelambakkam, Chennai, TN-603103, India; 2 Medical Bionanotechnology Lab, Department of Obstetrics and Gynaecology, Centre for Global Health Research, Saveetha Medical College, Saveetha Institute of Medical and Technical Sciences, Thandalam, Chennai, 602105, India; 3 Department of Radiology, Chettinad Hospital & Research Institute (CHRI), Chettinad Academy of Research and Education (CARE), Kelambakkam, Chennai, TN-603103, India

**Keywords:** Manganese ferrite nanoparticles, theranostics, carbon dots, silver nanoparticles, lipid nanoparticles, magnetic nanoparticles, multimodal diagnosis, cancer therapy

## Abstract

**Background and purpose:**

Multifunctional nanoparticles (NPs) are gaining significant interest in biomedical research because of their versatility and potential across various applications, especially in cancer imaging and therapy. These composite systems consist of assorted materials, such as metallic elements, metal oxides, polymers, and carbon-based nanostructures, that are combined to form a single platform featuring improved and synergistic properties.

**Experimental approach:**

This study aims to design and synthesize a novel class of silver-coated carbon dot-capped manganese ferrite NPs that were functionalized with lipids (L-Ag@MnFe@C) to improve their cytocompatibility and enable cancer therapy with multimodal imaging functionalities. Carbon-capped manganese ferrite NPs (MnFe@C) were synthesized by a one-pot hydrothermal method, followed by the fabrication of nano-silver coated over the surface (Ag@MnFe@C) using a modified Tollens method, and lipid functionalization was done by the rotary evaporation method for the development of low-cost and biodegradable theranostic agents.

**Key results:**

The physicochemical characterization reveals that the engineered L-Ag@MnFe@C exhibits a higher stability, with a zeta potential of -50.6 mV, a hydrodynamic diameter of 279.4 nm and a quantum yield of 69.4 %. The engineered NPs exhibit contrast capabilities in longitudinal magnetic resonance imaging, transverse magnetic resonance imaging, fluorescence imaging, and computed tomography imaging. Furthermore, L-Ag@MnFe@C demonstrated excellent anticancer activity on the lung cancer cell line (A549).

**Conclusion:**

Based on these studies, it can be concluded that the engineered L-Ag@MnFe@C exhibits multimodal imaging abilities and demonstrates anticancer properties, thereby confirming it as a potential theranostic agent.

## Introduction

Cancer is a malignant growth characterized by abnormal cell differentiation and rapid proliferation, loss of normal growth control, invasion into surrounding tissues, and the ability to spread to other parts of the body. Its development involves complex biological processes [1,2]. It remains a leading cause of illness and death in many regions worldwide, regardless of the country's development level. Cancer treatment has garnered significant focus in biomedical research over recent decades due to the grave danger it poses to human health [3,4]. The death rate from cancer rises each year, necessitating the creation of more effective therapeutic strategies. Despite major advancements in cancer therapy, it remains a considerable challenge because of issues with tolerance and compliance [5,6]. Theranostics is a term defined as a combination of therapeutic and diagnostic techniques most suitable for specific illnesses [7]. Theranostics highlights a close link between diagnostics and subsequent treatment, and this principle has gained substantial interest in personalized medicine, especially in oncology [8-10]. This approach has enabled the precise treatment of advanced-stage tumours with fewer adverse effects.

Theranostic agents such as radiotracers, liposomes, quantum dots, and plasmonic nanobubbles can be linked to anticancer medications, imaging compounds, and tumour cell markers with the aid of imaging techniques, offering the potential to enhance the diagnosis, treatment, and management of cancer patients [11-13]. Recently, nanoparticles (NPs) have been extensively researched and gradually developed as an alternative approach for cancer therapy, holding significant promise to aid in the diagnosis and treatment of tumours. Nanomaterials range in diameter from 1 to a couple of hundred nanometers and exhibit several unique characteristics that set them apart from small particles or bulk substances [14-16]. Due to their large specific surface area, enhanced surface activity, strong antioxidant capabilities, excellent biocompatibility, and solubility for molecular alterations, nanomaterials have significant potential for various biological applications [17]. These features make them particularly valuable in cancer treatment. Commonly used in medical applications are liposomes, carbon nanotubes, polymeric micelles, graphene, quantum dots, metallic nanoparticles, and magnetic nanoparticles. They are capable of efficiently penetrating dense tumour tissues [18-20].

Currently, numerous imaging technologies are utilized in the medical field, including computed tomography (CT), magnetic resonance imaging (MRI), ultrasound (US), single-photon emission computed tomography (SPECT), positron emission tomography (PET), fluorescence imaging, and Raman spectroscopy. In clinical medical imaging, contrast agents are typically used to improve image clarity, enabling differentiation between healthy and abnormal/tumour tissues [21,22]. Over recent years, MRI and CT have become essential diagnostic tools in clinical practice due to their notable advantages, such as excellent resolution, superior soft tissue contrast, and the ability to penetrate deep into tissues [8,23]. MRI contrast agents generally function by changing the relaxation times of water proton signals and are categorized into two types: longitudinal relaxation (*T*1) agents, which produce positive contrast images, and transverse relaxation (*T*2) agents, which generate negative contrast images [24]. Unlike conventional paramagnetic gadolinium, complex contrast agents like DOTAREM, Magnevist, ProHance, and superparamagnetic nanoparticles are increasingly becoming important as the next wave of magnetic probes due to their exceptional magnetic properties and extended circulation times [25-27].

In terms of design and usage, iron oxide nanoparticles have achieved notable advances in various biomedical applications; however, their inherent inability to produce positive contrast has restricted their use in MR imaging [28,29]. For *T*1 imaging, manganese chelates have become a promising alternative contrast agent. Haribabu *et al*. [30] synthesized ultrasmall manganese ferrite nanoclusters (PMNCs) with a diameter of 4 nm, which exhibit excellent *T*1 and *T*2 relaxation effects in MR imaging. They compared these synthesized PMNCs to commercially available Gd-DOTA and found that longitudinal molar relaxivity was 2.22 times better than Gd-DOTA. Additionally, the ratio of transverse (*r*2) to longitudal (*r*1) relaxivities (4.01) suggests that it is a promising candidate for both *T*1 and *T*2 MRI contrast agents. Recently, Saeidi *et al*. synthesized Mn-Zn ferrite (MZF) NPs that exhibited high relaxivities (*r*1 and *r*2) and a moderate *r*2/*r*1 ratio, suggesting that MZF is a promising candidate for negative contrast imaging. The NPs demonstrated increased *r*1 and decreased *r*2 values, making them suitable for applications as a dual-mode MRI contrast agent [31]. The biomedical applications of manganese ions are hampered by challenges, primarily due to their potential toxicity, limited biocompatibility, and diminished catalytic activity. While manganese plays a vital role in physiological functions, excess Mn^2+^ can generate oxidative stress, leading to cellular damage and neurotoxicity [32]. To overcome this issue, a protective coating or shell is engineered to improve biocompatibility without affecting its magnetic and catalytic performance.

Creating a protective coating with non-magnetic materials like carbon or silica on magnetic NPs will effectively overcome the key challenges [33-35]. Coating magnetic materials with carbon enhances their chemical and mechanical stability, making them durable against fluctuations in pH and temperature within cellular environments [36,37]. In addition to preserving magnetic functions, carbon coatings impart excellent optical qualities, aiding in the development of fluorescence-based imaging agents and biosensors. Due to their biocompatibility, resistance to photobleaching, ease of production, and customizable excitation/emission properties, carbon dots (CDs) stand out as a highly promising fluorescent material for medical and biological uses [38,39]. Sharmiladevi *et al*. [40] developed CDs decorated with manganese ferrite NPs to serve as a cost-effective and less-toxic multimodal contrast agent for fluorescence and MR imaging, aiming to replace conventional, heavy metal-based contrast agents. The cytotoxicity of both bare manganese ferrite NPs and CDs-coated manganese ferrite NPs was assessed in melanoma cell lines. Results show that manganese ferrite NPs coated with CDs (≈ 94 % cell viability) exhibited significantly higher biocompatibility compared to uncoated manganese ferrite N Ps (≈ 42 % cell viability). This indicates that the carbon coating sustainably mitigates the toxic effect of manganese ions, thereby enhancing the biocompatibility of NPs [40,41]. NPs coated with carbon possess oxygen vacancies on their surfaces, which enhance their absorption capabilities in the visible spectrum. Additionally, carbon coatings can diminish surface uniaxial anisotropy and lower the overall blocking temperatures [42,43]. As a result, the synthesized nanoparticles are often modified with various surface-stabilizing agents through surface modification techniques, which have gained popularity recently.

Silver nanoparticles (AgNPs) are among the most intriguing metallic nanomaterials, extensively utilized across multiple fields due to their remarkable properties [44,45]. Nanosilver-based structures are among the most extensively studied and researched in nanotechnology because of their significant surface area-to-volume ratio, chemical stability, catalytic properties, localized surface plasmon resonance, and high electrical conductivity [46]. Low-molecular-weight iodinated compounds like iopamidol and iodixanol are the most frequently employed contrast media in CT scans [47,48]. Iodine-based contrast agents are increasingly associated with side effects and are typically linked to allergic reactions and restrictions, particularly in individuals with kidney impairment and rapid elimination from the body [49]. To address these limitations, metal NPs-based formulations have emerged as a key area of focus, aiming to overcome the drawbacks associated with iodinated contrast agents in CT. The applications of Ag NPs in CT imaging contrast agents, along with other biomedical uses, have been widely studied. These materials exhibit unique and promising characteristics that make them suitable for a broad spectrum of biomedical applications [50-52]. Recently, they attracted significant interest because of their therapeutic advantages in diagnostic tools, wound healing, implants, broad-spectrum antibacterial agents capable of combating antibiotic-resistant bacteria, and as potential anticancer agents across various malignant cell lines [53]. The metal layer also protects the underlying carbon and iron oxide core from degradation or oxidation in biological environments. Bare Ag NPs exhibit low stability and limited targeting ability, which makes them unsuitable for *in vivo* applications. To address these challenges, biocompatible materials and polymers are used to modify Ag NPs, enhancing their stability and targeting capabilities [54].

Lipid modification of the nanoparticle surface is an effective strategy to enhance biocompatibility, dispersibility, and uptake by cancer cells. Lipids are compatible biological molecules characterized by an amphiphilic architecture, making them ideal candidates for coating inorganic NPs [55, 56]. The stability of colloidal NPs can be enhanced by attaching charged molecules to their surface. These charged molecules create electrostatic repulsion between NPs, which helps prevent them from clumping together. Lipid nanocarriers offer certain benefits over polymeric NPs, such as improved biocompatibility, reduced toxicity of the materials employed, low costs, greater scalability, and higher encapsulation efficiency for highly lipophilic agents [57,58].

In this study, carbon dot-capped manganese ferrite nanoparticles were synthesised via the hydrothermal method, then coated with silver NPs by the modified Tollens method, and functionalized with lipid layer using the rotary evaporation method, and it was mentioned as Lipid-Ag@MnFe@C. This layered design creates multifunctional nanoparticles with broader and more potent applications in biomedicine. Various characterization methods were used to verify the synthesis, and the multimodal imaging capability was evaluated across multiple imaging techniques. Additionally, its therapeutic effectiveness was assessed through *in vitro* cytotoxicity, and anticancer activity was assessed using V79 and A549 cells.

## Materials and methods

Trichloroiron hexahydrate (FeCl_3_·6H_2_O) and manganese chloride tetrahydrate (MnCl_2_·4H_2_O) were procured from Sigma-Aldrich, India. Urea (CH_4_N_2_O), citric acid monohydrate (C_6_H_8_O_7_·H_2_O), silver nitrate (AgNO_3_), lecithin soya, ammonia solution, sodium chloride (NaCl), doxorubicin hydrochloride (DOX), MTT salt (C_18_H_16_BrN_5_S), Alamar blue, Dulbecco's modified Eagle medium (DMEM), methanol, chloroform, and dimethyl sulphoxide (DMSO) were purchased from Himedia Laboratories, Waltham, USA. All chemicals used were of analytical grade (≥99 %) and were utilized in their original form. Double-distilled water (d/w) was employed for all experiments.

### Synthesis of carbon dot-capped manganese ferrite nanodots

The carbon dot-capped manganese ferrite nanodots (MnFe@C) were fabricated via the hydrothermal method. In 50 mL d/w, 0.2 M ferric chloride and 0.1 M manganese chloride were dissolved with a mechanical stirrer. Then, 0.5 M urea and 0.31 M citric acid were added, and the mixture was stirred for 10 min to ensure complete dissolution. The mixture was transferred into a Teflon-lined autoclave designed for hydrothermal reactions. The autoclave was sealed to create an airtight environment and then heated to 160 °C for 12 hours. After reaction, the autoclave was allowed to cool naturally to room temperature (~30 °C). The resulting mixture was centrifuged at 13,500 rpm for 25 mins at ~30 °C to separate the unreacted materials. The solid pellet was then dispersed in sterile d/w. This process was repeated several times to purify the product, after which the final pellet was dried at 100 °C in a hot air oven, then ground into a fine powder. The resulting MnFe@C nanodots were stored for further analysis.

### Synthesis of silver-coated MnFe@C

Silver nanoparticles (Ag NPs) were fabricated over the surface of MnFe@C using a modified Tollens method to synthesise Ag NPs. Aqueous silver nitrate (5 mM) was prepared for 10 mL, and 40 μL of 5M NaOH was added under continuous stirring. Subsequently, 100 μL of NH_3_ (aq) was introduced, and the mixture was continued stirring for 1 hour to allow the formation of [Ag(NH_3_)_2_]^+^ (silver-ammonia complex). The 5 mg of synthesised MnFe@C was added to the resulting [Ag(NH_3_)_2_]^+^ solution and stirred for another hour. Subsequently, 0.5 mL of a 0.5 M C_6_H_12_O_6_ solution was introduced and continuously stirred for another 3 h, resulting in silver-coated carbon dot-capped manganese ferrite nanodots (Ag@MnFe@C). The final dispersion containing the Ag@MnFe@C was centrifuged at 5000 rpm for 10 minutes, and this process was repeated twice to eliminate the unreacted molecules. Finally, it was dispersed in 10 mL of d/w and then used for further experiments.

### Synthesis of lipid-functionalized Ag@MnFe@C

Lipid functionalized Ag@MnFe@C (L-Ag@MnFe@C) were synthesised by dissolving 20 mg of soy lecithin in 3 mL of chloroform, and 5 mg of Ag@MnFe@C was mixed with 2 mL of methanol. Both mixtures were transferred to a round-bottom flask and subjected to vacuum-assisted rotary evaporation to produce a thin layer. After drying, the flasks were placed in a desiccator containing silica gel for 24 h to ensure complete removal of residual solvents. The resulting thin film was subsequently hydrated with 5 mL of d/w and sonicated for 15 min in a bath sonicator to obtain L-Ag@MnFe@C.

### Characterization of MnFe@C, Ag@MnFe@C, and L-Ag@MnFe@C

An FTIR/ATR spectrometer (Bruker-Alpha, USA) was used to identify the functional groups in the synthesized NPs. Absorbance measurements were performed with a Shimadzu UV-1800 spectrophotometer, Japan. The Jasco FP-3800 spectrofluorometer (Japan) measured the fluorescence excitation and emission spectra. The hydrodynamic diameter (dH) and zeta potential were analysed with a Malvern Nano-ZS-90 nanoparticle size analyser, UK, utilizing the principles of dynamic light scattering. The surface composition and chemical state of the NPs were analysed by Thermo Scientific Multilab 2000 X-ray photoelectron spectroscopy (XPS), USA. The magnetic properties of the synthesized nanoparticles were measured using the Lakeshore VSM 7410S, USA. A Bruker Single Crystal KAPPA APEXII X-ray diffractometer from the USA was employed to study the crystal structure. The NPs' morphology, structure, and size were examined using Philips JEM-2000EX TEM (Japan) and FEI Quanta FEG200F SEM (US). Phantom MR imaging was performed using a GE Signa HDxT 1.5 Tesla MRI scanner. Phantom CT images were captured with a Philips 64-slice CT scanner from the USA. Phantom fluorescence imaging was conducted on a PerkinElmer IVIS-Lumina LT small-animal imaging platform from the USA. The Olympus BX-51 fluorescence microscope from Japan was used to capture fluorescent cellular images.

### Stability assessment

The durability of the synthesized MnFe@C, Ag@MnFe@C, and L-Ag@MnFe@C was assessed using a spectrofluorimeter and zetasizer at various storage temperatures of 4 and ~30 °C. The evaluations were performed every 15 days over a period of 60 days to monitor variations in particle size distribution, surface charge, and fluorescence intensity.

### In vitro biocompatibility assessment

The cytocompatibility of the synthesized L-Ag@MnFe@C and Ag@MnFe@C NPs was evaluated in comparison to uncoated MnFe@C by treating it with fibroblast cells (V79), and then metabolic activity was measured. Briefly, V79 cells were cultured in 96-well plates at a density of 16,500 cells per well in complete DMEM and incubated overnight at 37 °C in a humidified atmosphere containing 5 % CO_2_ to promote cell adherence. Next, the culture medium in each well was replaced with fresh medium containing MnFe@C, Ag@MnFe@C, and L-Ag@MnFe@C at varying concentrations (5, 10, 15, 20, and 25 μM) and incubated for 24 h. Untreated cells served as the control group. Afterward, the medium in each well was replaced with a mixture of 10 vol.% of Alamar Blue reagent (0.15 mg/mL) and complete culture medium. The plates were then incubated at 37 °C for an additional 4 hours, and the fluorescence of Alamar Blue was recorded at an excitation wavelength of 530 nm and an emission wavelength of 584 nm using a Jasco FP-3800 plate reader. The amount of viable cells (Cell viability, %) was plotted against increasing concentrations of the NPs, Equation (1).



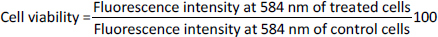

(1)


### Phantom MR imaging

Phantom MRIs were prepared with increasing concentrations (0.5 to 2.5 mM) of the synthesized MnFe@C, Ag@MnFe@C, and L-MnFe@C in a 96-well plate. These phantoms were then imaged using a *T*1-weighted FLAIR sequence to acquire 2 mm thick slices, with parameters: TR = 3000 ms, TE = 14 ms, FOV = 18×15 mm^2^, and various inversion times (TI) ranging from 400 to 2000 ms. For *T*2-weighted imaging, a turbo spin echo sequence was employed to obtain 2 mm slices with the following parameters: TR = 10,000 ms, TE varied between 10 and 100 ms, FOV of 18×15 mm^2^, and an echo train length of 12 [59]. The obtained slices were analysed using DICOM and ImageJ software to measure pixel intensities from each well. These data points were plotted to calculate the molar relaxivities (*r*1 and *r*2) of the engineered NPs. The ratio of *r*2 to *r*1 (*r*2/*r*1) was then determined to evaluate the contrasting effectiveness of L-Ag@MnFe@C for *T*1 and *T*2 imaging modalities.

### Phantom CT imaging

Conversely, the prepared phantom was exposed to X-ray radiation using a Philips CT Scanner. The imaging was performed with 150 mA tube current, 80 kVp (kilovolt peak) tube voltage, 3 mm slice thickness, 53×151 mm FOV, and 150 ms per rotation of exposure duration. The obtained slices were analysed using ImageJ software to measure pixel intensities from each well. These data points were plotted to calculate the average attenuation value of the engineered NPs to assess their ability to produce contrast in CT imaging.

### Phantom fluorescence imaging

The same phantom was examined in the PerkinElmer IVIS Lumina-LT animal imaging system to analyse the fluorescence imaging properties of the MnFe@C, Ag@MnFe@C, and L-Ag@MnFe@C NPs. Fluorescent images were captured utilizing an excitation filter of 535 nm, with emission detected within the 515 to 575 nm range. The resulting images were processed using the Living Image Software provided by the manufacturer.

### In vitro anti-tumour assessment

The anticancer effect of the synthesized Ag@MnFe@C and L-Ag@MnFe@C was evaluated by comparing it with an equivalent concentration of MnFe@C and free doxorubicin (DOX). This was carried out by treating A549 (human lung cancer) cells. The cells were seeded at a density of 23,500 per well in 48-well plates containing DMEM complete medium. They were cultured in a humidified incubator with 5 % CO_2_ at 37 °C for 24 h. Subsequently, the cells were treated with the same concentration of NPs used for toxicity assessment and further incubated for 24 h. After treatment, 50 μL of MTT reagent (5 mg/mL) was added and incubated in the dark for 4 h. To solubilize the formed formazan crystals produced by viable cells, DMSO was added and gently mixed. The optical density (OD) was then measured at 570 nm.

### Live/dead cell assay

A dual staining protocol was employed to assess cell viability, and fluorescent images of both living and dying cells were captured. In a coverslip, V79 and A549 cells were cultured in complete DMEM medium and incubated for 24 h at 37 °C under humidified conditions. Afterwards, the cells were treated with 25 μM of MnFe@C, Ag@MnFe@C, and L-Ag@MnFe@C NPs and then further incubated for another 24 h. The coverslip was placed on a clean, grease-free, and sterile glass slide, to which a mixture of acridine orange (AO) and ethidium bromide (EtBr) (each at 100 μg ml^-1^) dyes was added, followed by an incubation at 37 °C for 3 min. The stained cells were then observed and photographed under a fluorescent microscope using a suitable filter. The percentage of dead cells was calculated using the Equation (2).



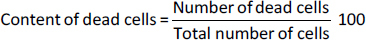

(2)


## Results and discussion

### Physicochemical characterizations

X-ray diffraction (XRD) analysis was systematically used to characterize the crystalline structure and phase composition of the synthesized MnFe@C and Ag@MnFe@C NPs. The distinct peaks at 2*θ* values of 31.6, 35.7, 48.6 and 56.3° correspond to (200), (311), (511), and (440), which are indicative of the spinel structure of manganese ferrite (MnFe_2_O_4_), aligning with the standard reference (ICDD PDF: 10-0319). The 35.7° peak is generally the most prominent in spinel ferrites, signifying the presence of a well-formed and well-crystallized ferrite phase, and other reflections (31.6°, 48.6°, and 56.3°) support the formation of the cubic spinel structure of manganese ferrite. The pattern exhibited a reflection that reached its maximum at 22.02° (002), indicating the presence of the turbostratic carbon phase of graphite, characterized by a structural arrangement of both amorphous and crystalline graphite components. This XRD pattern confirms the successful synthesis of MnFe NPs capped with carbon dots. The diffraction pattern for Ag@MnFe@C displays three supplementary peaks at 37.1°, 64.2°, and 77.1°, in addition to the other peaks observed in MNF@C, which are correspond to the (111), (220), and (300) planes of face-cantered cubic (fcc) silver (Ag), in agreement with ICDD PDF: 04-0783 confirming the successful decoration or coating of the NPs with metallic silver. Overall, the XRD results confirm the successful fabrication of both MnFe@C and Ag@MnFe@C NPs with well-defined crystalline MnFe_2_O_4_ cores, amorphous carbon dot shells, and additional silver phases in the latter.

**Figure 1.**
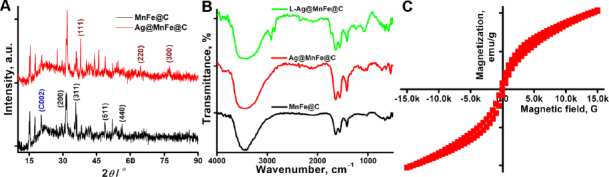
(A) XRD spectra of MnFe@C and Ag@MnFe@C, (B) FTIR spectra of MnFe@C, Ag@MnFe@C, and L-Ag@MnFe@C, (C) VSM image of L-Ag@MnFe@C

The FTIR spectra of MnFe@C, Ag@MnFe@C, and L-Ag@MnFe@C were recorded from 500 to 4000 cm^-1^ using KBr as a reference, to examine their chemical compositions and surface functional groups (Figure 1B). The broadband at 3470-3399 cm^-1^ corresponds to the O-H stretching vibration of hydroxyl groups. The 1560 and 1414 cm^-1^ bands are associated with asymmetric and symmetric stretching of C=C in graphitic domains. The absorption band at 1614 cm^-1^ for C=O stretching and 1016 cm^-1^ for C-O stretching vibration indicates the presence of carboxylic groups, suggesting that carbon dots were formed through the surface oxidation of citric acid. The bands at 657, 586, and 517 cm^-1^ are characteristic of metal-oxide stretching vibrations in the spinel ferrite structure. The Fe-O stretching band at 586 cm^-1^ indicates the strong signature of ferrite nanostructures. All the observed FTIR bands support the synthesis of MnFe@C NPs. With the introduction of silver in the Ag@MnFe@C NPs, similar functional groups were observed. A new band at 639 cm^-1^ suggested Ag-O interactions, while a distinctive peak at 1639 cm^-1^ indicated silver attachment, confirming the development of a coating on the surface of MnFe@C. The band at 2925 and 2855 cm^-1^ corresponds to the asymmetric and symmetric stretching of -CH_2_ groups, which strongly suggests the presence of long hydrocarbon chains in the lipid. The strong band at 1729 cm^-1^ is attributed to C=O stretching of ester groups, confirming the successful functionalization of the lipid. These significant FTIR bands confirm the lipid-functionalized Ag@MnFe@C NPs.

The magnetic characteristics of the synthesized L-Ag@MnFe@C were examined using VSM to confirm their potential use in MR imaging (Figure 1C). The results revealed a near-zero hysteresis loop, confirming that the synthesized magnetic particles exhibit superparamagnetic behaviour at ambient temperature, with a minute amount of ferromagnetism due to thermal fluctuations, thus making them highly advantageous for use as MRI contrast agents. The S-shaped magnetization curve passes through the origin, exhibiting minimal residual magnetization (Mr) and coercive field (Hc), indicating that once the external magnetic field is removed, the magnetization returns to zero. This signature behaviour is characteristic of superparamagnetic material. The superparamagnetism suggests that the optimized hydrothermal synthesis method can produce highly crystalline, phase-pure NPs of L-Ag@MnFe@C, demonstrating that the silver coating and lipid functionalization do not affect the magnetic behaviour of the MnFe core.

**Figure 2.**
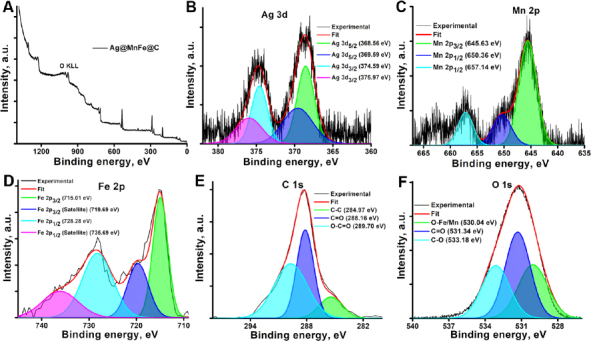
XPS spectral image of Ag@MnFe@C (A), and deconvoluted spectra of Ag 3d (B), Mn 2p (C). Fe 2p (D), C 1s (E), and O 1s (F)

X-ray photoelectron spectroscopy (XPS), a versatile surface analysis technique, is commonly employed to examine the composition and chemical states of materials. In-depth surface investigations of the nanoparticles were conducted using XPS to determine the chemical states of each element in the Ag@MnFe@C (Figure 2A). The Ag 3d, Mn 2p, Fe 2p, C 1s, N 1s, and O 1s photoelectron spectra of the sample Ag@MnFe@C are shown in Figure 1B-F. High-resolution XPS spectra of the Ag 3d doublet displayed an asymmetrical peak featuring two spin-orbit components at binding energies of 368.56 eV (Ag 3d_5/2_) and 374.59 eV (Ag 3d_3/2_), separated by 6 eV (Figure 2B). These characteristics clearly confirmed the presence of metallic Ag nanoparticles on the surface. Furthermore, spectral deconvolution revealed additional peak pairs at 369.59 eV (Ag 3d_5/2_) and 375.97 eV (Ag 3d_3/2_), indicating the existence of silver in the +1 oxidation state. Two asymmetric peaks observed around 645.63 and 657.14 eV (Figure 2C) correspond to Mn 2p_3/2_ and Mn 2p_1/2_, respectively.

The deconvolution revealed the additional peak at 650.36 eV (Mn 2p_1/2_). As shown in Figure 2D, the Fe 2p peaks consist of two primary asymmetric components with the binding energies of 715.01 eV (Fe 2p_3/2_) and 728.28 eV (Fe 2p_1/2_), and the doublet separation between them is 13 eV. Additionally, two smaller peaks around 719.69 and 735.69 eV are satellite features associated with Fe 2p_3/2_ and Fe 2p_1/2_, respectively. For the Fe 2p_3/2_, the peaks at 715.01 and 719.69 eV correspond to the Fe^3+^ cations occupying either tetrahedral or octahedral sites within the spinel ferrite structure. The high-resolution XPS spectra show peaks at 288 and 533 eV, which correspond to the 1s orbitals of carbon and oxygen atoms, respectively, indicating the presence of C and O in the synthesized NPs. The deconvoluted spectra reveal three peaks in Figure 2E at 284.97, 288.16 and 289.70 eV, attributed to the different oxidation states of carbon: C-C, C=O, and O-C=O, respectively. The XPS spectrum of the O 1s, depicted in Figure 2F, displays three distinct oxygen-related peaks at binding energies of 530.04, 531.34 and 533.18 eV. The peak at 530.04 eV is indicative of lattice oxygen involved in metal-oxygen bonds, associated explicitly with O-Fe and O-Mn linkages within the spinel crystal structure of MnFe_2_O_4_, confirming the existence of the ferrite phase. The signal at 531.34 eV (C=O) and 533.18 eV (C-O) is assigned to oxygen atoms bonded to carbon, originating from oxygen-containing functional groups present on the carbon coating. These results established that the synthesised CDs-capped MnFe NPs were coated with silver NPs.

### Optical properties

**Figure 3.**
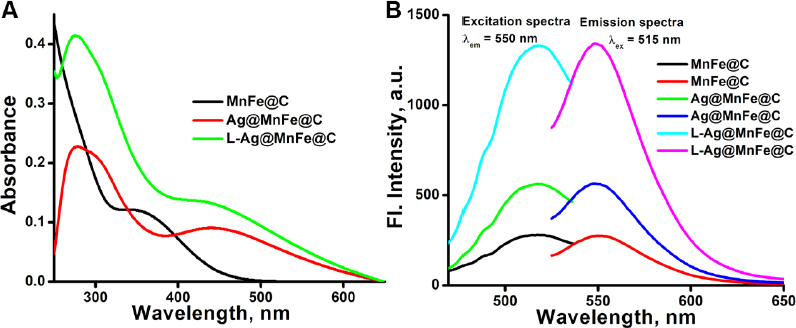
**A)** Absorption spectra of MnFe@C, Ag@MnFe@C, and L-Ag@MnFe@C. **(B)** Steady-state fluorescence spectra of MnFe@C, Ag@MnFe@C, and L-Ag@MnFe@C.

The optical properties of the synthesized MnFe@C, Ag@MnFe@C, and L-Ag@MnFe@C were examined using UV-Vis absorption and steady-state fluorescence spectroscopy. The absorption spectra of the synthesized MnFe@C nanoparticles displayed a weak π→π* transition peak with a broad range between 250 and 280 nm, and a sharp peak of n→π* transition at 357 nm attributed to the formation of carbon dots (CDs) (Figure 3A). The weaker π→π* transition indicates the carbon dot layer lacks extended domains [60]. Some minor absorption peaks were also observed in the range of 480 nm to 520 nm. The Ag@MnFe@C shows a border peak of 405 to 470 nm, centered at 430 nm, corresponding to Ag NPs surface plasmon resonance (SPR) arising from collective electron oscillation of the metal NPs. This confirms the successful synthesis of silver coating and aligns with Ag NPs SPR wavelengths (400 – 450 nm). Another peak at 275 nm is responsible for the π→π* transition, which is due to the silver deposition, which promotes larger sp^2^ domains in the carbon shell, enabling stronger π→π* transitions. The plasmonic field of Ag enhances the local electromagnetic field, increasing the oscillator strength of this transition in the carbon layer by the surface plasmon coupling effect [61,62]. The presence of Ag might introduce plasmonic effects that overlap with the n→π* region and mask the peak. Overall, these confirm the nano silver coated over the MnFe@C. The lipid functionalized Ag@MnFe@C showed similar absorption features, with enhanced absorption, which is possibly due to modifications in its microenvironment surrounding the Ag@MnFe@C NPs.

The steady-state fluorescence spectra of the MnFe@C, Ag@MnFe@C, and L-Ag@MnFe@C NPs are displayed in Figure 3B. The synthesized nanoparticles exhibited interesting emission properties. All three engineered particles showed an emission peak (*λ*_em_) at 550 nm. They displayed a sharp peak at 515 nm when the excitation spectra were recorded with respect to *λ*_em_ = 550 nm. Therefore, 515 nm was fixed as the excitation wavelength for all emission studies. In contrast, when the synthesized MnFe@C was excited at 515 nm, a faint emission peak was observed at 550 nm with an intensity of 274.6 a.u., and its quantum yield was found to be 18.8 %. The Ag@MnFe@C exhibits a higher emission intensity of 561.2 a.u. at the same emission wavelength of 550 nm for the same concentration, which was almost twice the intensity of MnFe@C. The Ag exhibits strong localized SPR, which amplifies the local electromagnetic field near its surface, increasing the fluorescence rates. When Ag NPs are coated onto MnFe@C, the plasmonic near-field interacts with the carbon dots, accelerating radiative decay and increasing the quantum yield by 42.1 %. This reduces non-radiative pathways, leading to higher emission intensities [63]. Meanwhile, the L-Ag@MnFe@C demonstrated a more prominent emission peak at 550 nm, with a notably increased intensity of 1336.3 a.u., under the same excitation wavelength (515 nm), which showed 4.8 times higher intensity than MnFe@C. The lipid layer functionalization creates a hydrophobic protective layer that shields surface defects, alters the local dielectric constant, prevents nanoparticle (NP) aggregation, and maintains monodispersity [64]. These effects minimize non-radiative decays and consequently enhance the quantum yield to 69.4 %. This was further confirmed by excitation spectra, where the emission wavelength was held constant at 550 nm, producing a fluorescence peak at 515 nm (Figure 3B).

### Particle size analysis

**Figure 4.**
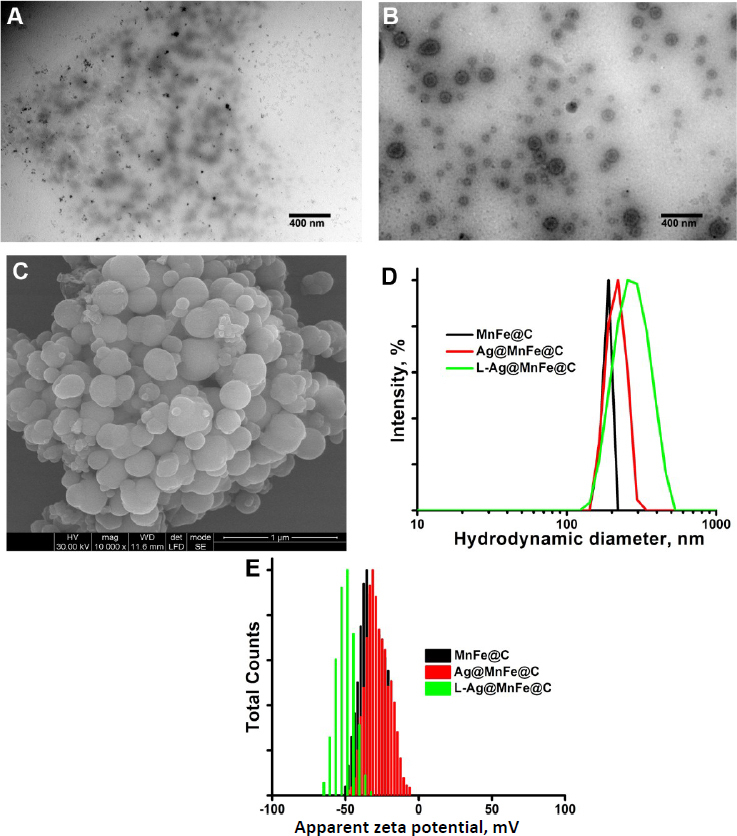
(A) TEM of MnFe@C, (B) TEM image of L-Ag@MnFe@, (C) HRSEM of L-Ag@MnFe@C, (D) Hydrodynamic diameter of MnFe@C, Ag@MnFe@C, and L-Ag@MnFe@C, (E) Zeta potential of MnFe@C, Ag@MnFe@C and L-Ag@MnFe@C

The surface morphology and crystal arrangement of the synthesized MnFe@C and Ag@MnFe@C NPs were investigated using HRSEM and TEM. It will help to assess the morphological uniformity, size distribution, surface features, and structural integrity of engineered NPs. The TEM image shows that the synthesised MnFe@C is uniformly distributed and primarily exhibits a spherical nanoparticle shape (Figure 4A). The average particle size was found to be 86.8 ± 7.5 nm, which is particularly suitable for biomedical applications. The TEM images of L-Ag@MnFe@C (Figure 4B) clearly demonstrated a well-defined core-shell nanostructure. The magnetic core located at the centre of the particles is surrounded by a shell composed of silver and lipid layers. This configuration confirms the successful sequential assembly of silver over MnFe@C, followed by lipid functionalization. The average size of the L-Ag@MnFe@C was 143.7 ± 12 nm. The increase in particle size compared to MnFe@C confirms the presence of multiple surface layers. The HRSEM image of L-Ag@MnFe@C shows a uniformly dispersed and spherical morphology, which is attributed to the lipid functionalization over Ag@MnFe@C. This functionalization enhances the structural stability and surface uniformity of the composite material (Figure 4C).

The colloidal characteristics were assessed using a particle size analyser based on the dynamic light scattering technique. The colloidal stability and particle size distribution play a critical role in determining the efficacy, dispersibility, and long-term usage of NPs in biomedical applications. The hydrodynamic diameter (dH) for MnFe@C was measured as 189.3 nm, while the dH of Ag@MnFe@C was increased to 214.4 nm. The increase in hydrodynamic size is attributed to the successful coating of silver on the surface. Further functionalization with a lipid molecule (L-Ag@MnFe@C) increases the size to 279.4 nm (Figure 4D). The polydispersity index (PDI) is a dimensionless parameter that quantifies the distribution width of particle sizes in a colloidal dispersion.

PDI values range from 0 to 1, where lower values (<0.01) denote a monodisperse system with uniform particle sizes, and higher values (above 0.7) indicate a polydisperse system. The PDI of MnFe@C was observed as 0.729, suggesting a nearly heterogeneous particle size distribution. The Ag@MnFe@C exhibited a significantly improved PDI of 0.628, suggesting a more controlled growth mechanism during silver coating, although the system remained moderately polydisperse. Notably, the lipid-functionalized (L-Ag@MnFe@C) NPs demonstrated the lowest PDI of 0.223 (Table 1), indicating a relatively uniform particle size distribution within a physiologically acceptable range for biomedical applications. The improvement in PDI upon lipid functionalization is attributed to stabilization provided by the lipid layer, which inhibits aggregation and promotes more homogeneous dispersion in aqueous media. The zeta potential provides information about the surface charge of the NPs in an aqueous environment. Generally, NPs with zeta potential values greater than ±30 mV are considered stable due to the strong electrostatic repulsion between particles that prevents them from aggregating. The zeta potential of MnFe@C and Ag@MnFe@C was -36.1 and -31.3 mV, indicating moderately stable colloidal systems (Figure 4E) [65]. The small decrease in surface charge is due to the change in surface functional groups upon silver coating. The L-Ag@MnFe@C demonstrated the highest zeta potential of -50.6 mV, highlighting their superior colloidal stability, making them promising candidates for biomedical applications.

**Table 1.** Hydrodynamic diameter, PDI, zeta potential, and quantum yield of the MnFe@C, Ag@MnFe@C, and L-Ag@MnFe@C.

Nanoparticles	Hydrodynamic diameter, nm	PDI	Zeta potential, mV	Quantum yield, %
MnFe@C	184.9	0.729	-36.1	18.8
Ag@MnFe@C	214.4	0.628	-31.3	42.1
L-Ag@MnFe@C	279.4	0.223	-50.6	69.4

### Stability studies

**Figure 5.**
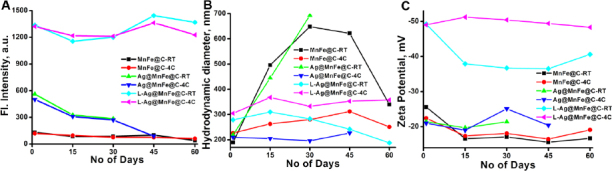
The stability analysis of MnFe@C, Ag@MnFe@C, and L-Ag@MnFe@C at varying storage conditions using fluorescence intensity (A), Hydrodynamic size (B) and surface charge (C)

Stability of the synthesised NPs was examined by several factors, including temperature, fluorescence properties (Figure 5A), size (Figure 5B), and surface charge (Figure 5C) over 45 days. For MnFe@C and L-Ag@MnFe@C, the particles remain stable at 4 °C with no significant change in fluorescence intensity, hydrodynamic diameter, and zeta potential, indicating good colloidal stability. At room temperature, MnFe@C showed variations, but L-Ag@MnFe@C remains the same. Ag@MnFe@C displayed instability by changes in their properties after 15 days at room temperature and 30 days at 4 °C, suggesting that silver coating plays a critical role in altering colloidal behaviour. The changes in surface chemistry due to silver oxidation in an aqueous environment promote aggregation. L-Ag@MnFe@C remains more stable, due to additional lipid functionalization providing a steric barrier that prevents agglomeration. Lipids enhance hydrophilicity and create a stabilizing corona, minimizing surface energy and protecting silver from oxidation. This suggests that storing the samples at 4 °C enhances their stability, making them more appropriate for long-term applications.

### Biocompatibility assessment

**Figure 6.**
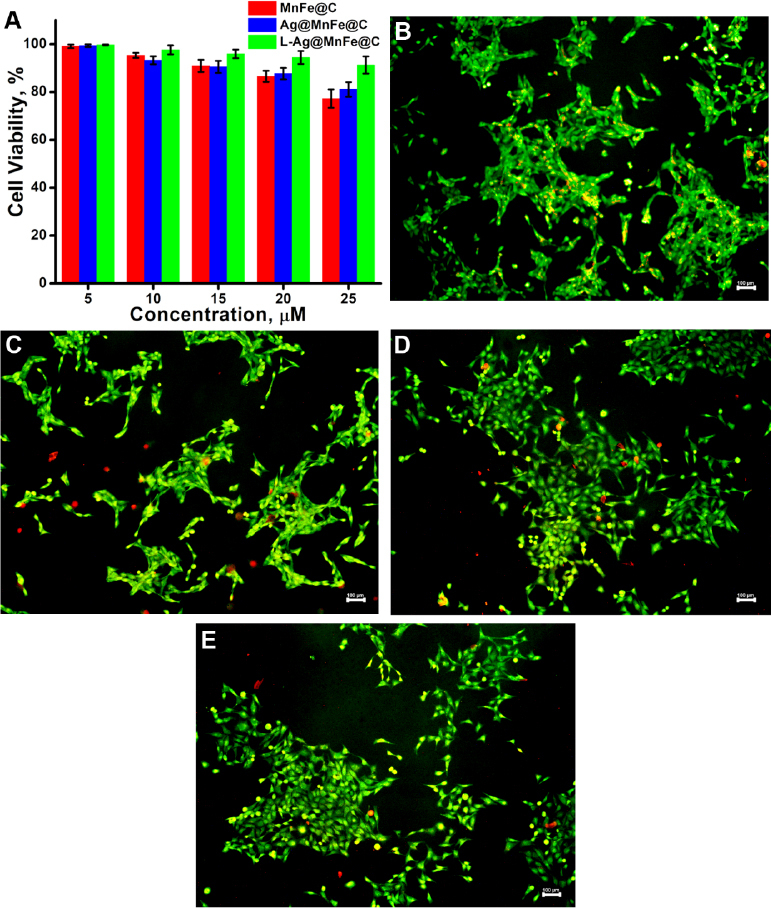
(A) Cell viability study using Alamar blue assay on V79 cells. Fluorescence microscopic images of V79 cells stained with AO/EtBr (10× magnification) of control (B), cells treated with 25 μM of MnFe@C (C), cells treated with 25 μM of Ag@MnFe@C (D) and cells treated with 25 μM of L-Ag@MnFe@C (E). (Scale bar = 100 (μm)

The cytotoxic effects of the synthesized MnFe@C, Ag@MnFe@C, and L-Ag@MnFe@C were evaluated using the Alamar blue assay on the V79 cell line (Figure 6A). The MnFe@C demonstrated 77.2 % cell viability at higher concentrations, suggesting low toxicity. The carbon coating enhances stability and improves biocompatibility by reducing direct interaction between the metal oxide core and the cellular environment.

The Ag@MnFe@C showed a similar cell viability of 81.2 %. This reduction is attributed to the silver component, which can induce oxidative stress, disrupt cellular membranes, or interfere with essential intracellular processes. Interestingly, the lipid-functionalized Ag@MnFe@C (L-Ag@MnFe@C) exhibited a significant improvement in cell viability, achieving 93.3 %. This suggests that lipid functionalization plays a crucial role in enhancing biocompatibility. Lipids can form a biocompatible layer that mimics the natural components of cell membranes, promoting better cellular interaction and reducing non-specific toxicity. A double-staining technique using AO/EtBr was employed for a live/dead cell assay, where live cells appeared green and dead cells appeared red. Figure 6B illustrates the control cells, which were entirely viable. Figures 6C, D, and E display the cells treated with 25 μM of MnFe@C, Ag@MnFe@C, and L-Ag@MnFe@C, respectively.

The MnFe@C treated cells exhibited a cell viability of 80 %, while the Ag@MnFe@C treated cells showed an 87 % viability. Notably, the L-Ag@MnFe@C treated cells demonstrated an improved viability rate of 91 %, compared with MnFe@C and Ag@MnFe@C. These results match MTT results, indicating that L-Ag@MnFe@C is suitable for theranostic applications.

### In vitro MR imaging

**Figure 7.**
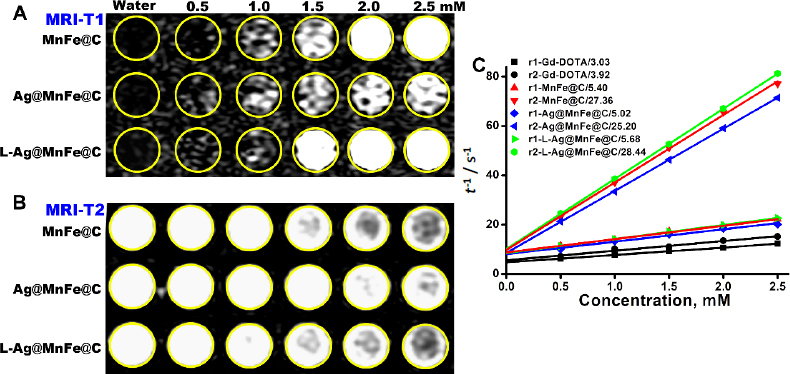
(A) *T*1-weighted phantom images of MnFe@C, Ag-MnFe@C, and L-Ag@MnFe@C, (B) *T*2-weighted phantom images of MnFe@C, Ag-MnFe@C, and L-Ag@MnFe@C, (C) The relaxivity data were plotted against the concentration of MnFe@C, Ag@MnFe@C, and L-Ag@MnFe@C and compared with commercially available Gd-DOTA

To assess the contrast effect of MnFe@C, Ag@MnFe@C, and L-MnFe@C on MRI, *T*1- and *T*2-weighted images were obtained from phantoms containing these NPs at concentrations ranging from 0.5 to 2.5 mM. These images were subsequently analysed and compared to each other as well as to water without any added NPs, which was taken as the control. Using DICOM image processing software, intensities were measured. The engineered NPs showed a positive contrast effect due to the presence of Mn in the structure of NPs (Figure 7A).

In contrast, while MnFe@C showed enhanced intensity at 2 mM, L-Ag@MnFe@C displayed better intensity at a lower concentration of 1.5 mM. The Ag@MnFe@C exhibits a very small contrast effect, likely due to the silver oxidation, which promotes aggregation. Lipid functionalized Ag@MnFe@C (L-Ag@MnFe@C) exhibits *T*1 contrast at lower concentrations (1.5 mM) compared to MnFe@C. Lipid layer prevents aggregation and ensures a higher surface-to-volume ratio, enhancing water proton access to the magnetic core. The engineered NPs exhibited a negative contrast effect due to the presence of Fe in their structure, which enhances *T*2-weighted MR imaging (Figure 7B). The MnFe@C and L-Ag@MnFe@C showed a reduction in intensity at 1.5 mM concentration, but Ag@MnFe@C showed a reduction at 2.0 mM concentration. From the MRI images, we found that the change in signal intensity (for both *T*1 and *T*2) was directly proportional to the concentration. To assess the contrasting effectiveness of the contrast agents, the longitudinal (*r*1 = 1/*T*1) and transverse (*r*2 = 1/*T*2) relaxivities are critically important. Graphs have been plotted showing the relationship between relaxivities and increasing concentrations, which helps in determining the molar relaxivities. The molar longitudinal relaxivity (*r*1) of MnFe@C was measured to be 5.40 mM^-1^ s^-1^, Ag@MnFe@C was 5.02 mM^-1^ s^-1^, and L-Ag@MnFe@C was 5.68 mM^-1^ s^-1^. The molar transverse (*r*2) relaxivity was found to be 27.36, 25.20 and 28.44 mM^-1^ s^-1^ for MnFe@C, Ag@MnFe@C, and L-Ag@MnFe@C. Similar concentrations of the commercially used contrast agent Gd-DOTA were applied to assess its relaxivity performance, showing that Gd-DOTA had *r*1 and *r*2 relaxivity values of 3.03 and 3.92 mM^-1^ s^-1^, respectively. These values were considerably lower than those of the engineered nanoparticles. The ratio of the molar relaxivities (*r*2/*r*1) of MnFe@C, Ag@MnFe@C, and L-Ag@MnFe@C was determined as 5.06, 5.01 and 5.00, which falls within the range of 10 > *r*2 to *r*1 > 3. In contrast, Gd-DOTA had an *r*2/*r*1 ratio of 1.29, which is not in the specified range. This suggests their suitability as a dual-mode MR contrast agent of the engineered NPs, capable of exhibiting both *T*1 and *T*2-weighted imaging.

### In vitro fluorescence imaging

**Figure 8.**
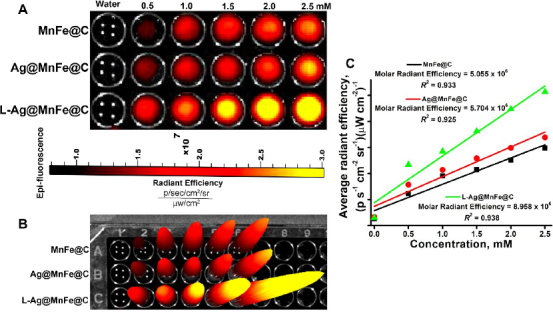
(A) Phantom optical images of MnFe@C, Ag-MnFe@C, and L-Ag@MnFe@C, (B) 3D phantom image of the MnFe@C, Ag-MnFe@C, and L-Ag@MnFe@C, (C) The optical image intensities were plotted against the concentration of MnFe@C, Ag-MnFe@C, and L-Ag@MnFe@C to determine the molar radiant efficiency

Figures 8A and B display the optical characteristics of MnFe@C, Ag@MnFe@C, and L-Ag@MnFe@C as fluorescent imaging agents in a small-animal imaging platform. All fluorescence levels were normalized to photons per second per square centimetre per steradian (p s^-1^ cm^-2^ sr^-1^), after subtracting the background signals. The results show that the fluorescence signal is proportional to the concentration. The MnFe@C exhibits fluorescence properties, and the radiant efficiency was found as 1.49×10^7^ p s^-1^ cm^-2^ sr^-1^ for higher concentration (2.5 mM). The fluorescence intensity of Ag@MnFe@C was increased to 1.59×10^7^ p s^-1^ cm^-2^ sr^-1^. The Ag coating introduces LSPR that amplifies the local electromagnetic field. The lipid functionalization leads to a more significant increase in fluorescence signal (2.57×10^7^ p s^-1^ cm^-2^ sr^-1^), up to a 65 % increment from Ag@MnFe@C to L-Ag@MnFe@C (1.59×10^7^ → 2.57×10^7^ p s^-1^ cm^-2^ sr^-1^). The functionalization passivates surface defects and improves the dispersability of NPs. Figure 8C presents a graphical representation of average radiative efficiency in relation to the concentrations of MnFe@C, Ag@MnFe@C, and L-Ag@MnFe@C. The slope indicates the mean radiative efficiency, which was determined to be 5.055×10^6^, 5.704×10^6^ and 8.958×10^6^ p s^-1^ cm^-2^ sr^-1^ mM^-1^, respectively. The data clearly showed that the lipid-modified Ag@MnFe@C exhibited higher radiant efficiencies in comparison to both Ag@MnFe@C and MnFe@C. These results suggest that L-Ag@MnFe@C is a promising candidate for highly sensitive fluorescence imaging, and it is in good agreement with the steady-state fluorescence analysis.

### *In vitro* CT imaging

**Figure 9.**
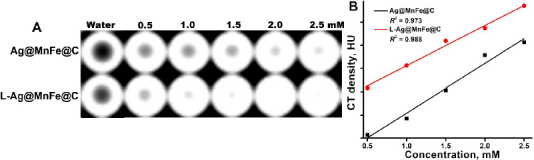
(A) Phantom CT image of the Ag@MnFe@C, and L-Ag@MnFe@C, (B) The CT image intensities (HU) were plotted against the concentration of Ag-MnFe@C and L-Ag@MnFe@C to determine the attenuation rate

The engineered NPs were designed to provide added benefit beyond the imaging capabilities of *T*1- and *T*2- MR imaging, and fluorescence imaging. CT imaging primarily relies on the attenuation of X-rays as they pass through an object from various angles. The contrast in CT images depends largely on the atomic number and the attenuation coefficient of the contrast agent employed. Metallic nanomaterials are extensively studied for their potential as CT contrast agents because they exhibit these properties. The nano-silver shell in the Ag@MnFe@C and L-Ag@MnFe@C is hypothesized to function as a contrast agent for CT imaging, similar to how gold NPs have been traditionally researched for this purpose. Figure 9A displays the phantom CT images obtained with increasing concentrations of Ag@MnFe@C and L-Ag@MnFe@C, as detailed earlier. The signal intensities in the phantom images were observed to increase proportionally with increasing Ag concentrations. These signal intensities were employed to assess the CT attenuation properties of the prepared NPs. Therefore, the engineered particles have potential as non-invasive contrast agents for CT imaging. The contrast provided by nano-silver is often observed to be approximately 2.5 times greater than that of iodine-based agents. Consequently, lower doses of Ag NPs can produce comparable levels of contrast to iodinated contrast agents. Although NPs possess advantageous properties, they tend to become unstable and clump together when exposed to strong counter surface charges and high salinity levels. Coating the silver shell with lipids (functionalization) has been recognized as an effective strategy to prevent destabilization and aggregation caused by saline environments or other restrictive conditions. As a result, L-Ag@MnFe@C exhibits greater stability than Ag@MnFe@C, leading to improved CT contrast performance, as illustrated in Figure 9A. The direct relationship between CT densities measured in HU and the calculated CT attenuation rates for both Ag@MnFe@C and L-Ag@MnFe@C was determined to be 321.53 ± 9.23 and 359.98 ± 7.37 HU/mg, respectively (Figure 9B). The measured CT attenuation rate was greater than that of previously studied Au NPs used in CT imaging. This indicates that the synthesized L-Ag@MnFe@C can serve as an effective contrast agents in multimodal imaging for twin-mode MRI, fluorescence imaging, and CT scans, aiding in distinguishing between normal and cancerous tissues.

### Anticancer activity

**Figure 10.**
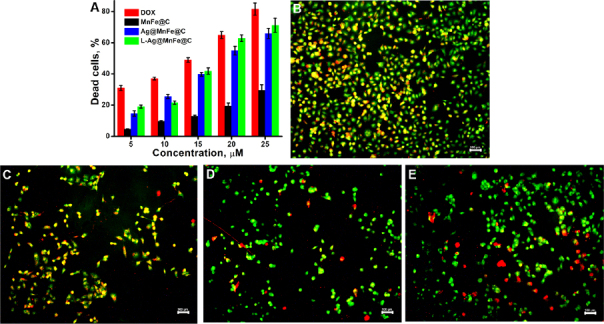
(A) Cell death percentage of DOX, MnFe@C, Ag@MnFe@C, and L-Ag@MnFe@C was evaluated using the MTT assay. Fluorescence microscopic images of A549 cells stained with AO/EtBr (10× magnification) of control (B), cells treated with 25 μM of MnFe@C (C), cells treated with 25 μM of Ag@MnFe@C (D), and cells treated with 25 μM of L-Ag@MnFe@C (E). (Scale bar = 100 μm)

An *in vitro* experiment was conducted on A549 cells to evaluate the anticancer activity by measuring the number of dead cells after treatment with DOX, MnFe@C, Ag@MnFe@C, and L-Ag@MnFe@C (Figure 10A).

The result shows dose-dependent cytotoxicity in A549 cells. At 25 μM, DOX achieves 86.6 % cell death, and MnFe@C shows lower cytotoxicity (29.5 %). This shows that MnFe@C does not have any anticancer activity. After coating with silver, the MnFe@C (Ag@MnFe@C) significantly increased cell death to 66 %. This substantial increase from 29.5 to 66 % killing indicates that Ag coating greatly improves anticancer efficacy. Ag NPs are known to induce ROS-dependent apoptosis. The L-Ag@MnFe@C demonstrated a cell death rate of 71.2 %. The enhanced therapeutic effectiveness is mainly due to the lipid functionalization on its surface, which significantly improves cellular uptake by promoting better interaction and fusion with cell membranes. A live/dead cell viability assay was conducted using a dual staining technique with acridine orange and ethidium bromide, where viable cells appear green and dead cells are stained red. Figures 10 B-E display the results for control cells, as well as cells treated with 25 μM of MnFe@C, 25 μM of Ag@MnFe@C and 25 μM of L-Ag@MnFe@C. In the control group, all cells are shown in green, indicating they are alive. Conversely, in the treated groups, the percentage of dead cells is 24.7, 66.4 and 69.9 %, respectively. The live/dead cell viability assay results match the previous data. These results show that Ag coating in MnFe@C significantly enhances the therapeutic effectiveness of the NPs, particularly for the nanoformulation without a loaded chemotherapeutic drug.

## Conclusion

In this study, lipid-functionalized silver-coated carbon dot-capped manganese ferrite (L-Ag@MnFe@C) NPs were synthesized to enhance theranostic efficacy with improved biocompatibility and reduced toxicity. Different characterization techniques were employed at each stage of particle development to confirm their composition, structure, and physicochemical characteristics. The XRD and XPS results clearly demonstrated the successful synthesis of silver NPs over the magnetic NPs. The TEM images distinctly show that the MnFe@C core is coated with an Ag shell, and the lipid layer was about 87.9 ± 8 nm in thickness. Phantom-based imaging experiments demonstrated that specially designed NPs are capable of producing *T*1- and *T*2-weighted MRI signals. The carbon layer gave the particles fluorescent capabilities, allowing for their use in fluorescence-based imaging techniques. The silver shell of the NPs enhanced their precision in fluorescence imaging and additionally contributed to the generation of CT signals. The surface modification of the Ag@MnFe@C with lipid enhances the accuracy in fluorescence imaging and achieves a superior quantum yield of 69.4 %. The cytocompatibility of the NPs was investigated on fibroblast cells, revealing that the lipid layer increases the biocompatibility of the synthesized NPs. The Ag layer provides the therapeutic efficacy, and it was investigated in lung cancer cells. The finding reveals that L-Ag@MnFe@C has similar anticancer activity compared to DOX. Overall, the specially designed L-Ag@MnFe@C NPs demonstrate exceptional sensitivity, biocompatibility, bioavailability, and affordability, making them a highly promising material for theranostic applications in multimodal imaging and cancer treatment.
